# MicroRNAs expression profile in chemotherapy-induced cardiotoxicity in non-small cell lung cancer using a co-culture model

**DOI:** 10.17305/bb.2023.9272

**Published:** 2024-02-01

**Authors:** Mihai Romitan, Oana Zanoaga, Liviuta Budisan, Ancuta Jurj, Lajos Raduly, Laura Pop, Cristina Ciocan, Radu Pirlog, Cornelia Braicu, Tudor Eliade Ciuleanu, Ioana Berindan-Neagoe

**Affiliations:** 1 Research Center for Functional Genomics, Biomedicine and Translational Medicine, Iuliu Haţieganu University of Medicine and Pharmacy, Cluj-Napoca, Romania; 2 Department of Oncology, Iuliu Haţieganu University of Medicine and Pharmacy, Cluj-Napoca, Romania; 3 Department of Oncology, Prof. Dr. Ion Chiricuta Oncology Institute, Cluj-Napoca, Romania

**Keywords:** Cardiotoxicity, lung cancer, carboplatin, vinorelbine, miR-21-5p, miR-30c-5p, miR-205-5p

## Abstract

Clinical application of chemotherapy in lung cancer is constrained by side effects, notably cardiotoxicity, the mechanisms of which remain elusive. This study assessed the potential of specific microRNAs (miRNAs) as biomarkers for chemotherapy-induced cardiotoxicity in lung cancer. We employed two lung adenocarcinoma cell lines (Calu6 and H1792) and ventricular normal human cardiac fibroblasts (NHCF-V) in single and co-culture experiments. Functional tests were conducted using 100 **µ**M carboplatin and 1 **µ**M vinorelbine doses. The effects of carboplatin and vinorelbine, both individually and in combination, were evaluated at cellular and molecular levels 48 h post-therapy for both mono- and co-cultures. miR-205-5p, miR-21-5p, and miR-30a-5p, modulated by anticancer treatments and influencing cardiotoxicity, were analyzed. Vinorelbine and carboplatin treatment promoted apoptosis and autophagy in lung cancer cells and cardiac fibroblasts more than in controls. Western blot analyses revealed BCL2 and p53 protein upregulation. Using qRT-PCR, we investigated the expression dynamics of miR-21-5p, miR-30c-5p, and miR-205-5p in co-cultured cardiomyocytes and lung cancer cells, revealing altered miRNA patterns from vinorelbine and carboplatin treatment. Our findings underscore the intricate relationship between chemotherapy, miRNA regulation, and cardiotoxicity, highlighting the importance of cardiac health in lung cancer treatment decisions.

## Introduction

Cardiovascular disease (CVD) and cancer consistently rank as the leading causes of morbidity and mortality worldwide [1]. CVD accounts for approximately 17.3 million deaths per year [2], while in 2020, cancer led to nearly 10 million deaths out of 19.3 million new diagnoses [3]. Although cardiotoxicity is a well-documented side effect of chemotherapy, the mechanisms underlying this phenomenon remain elusive. Notably, cardiotoxicity in cancer patients has been observed following treatments with drugs, such as anthracyclines and tyrosine kinase inhibitors [4]. Most cardiotoxic effects and cardiovascular complications manifest within the first year after a cancer diagnosis [3]. The clinical manifestations of cardiotoxicity include a large spectrum of disorders, from mild transient arrhythmias to infarction and cardiomyopathy [5]. Risk factors for chemotherapy-induced cardiotoxicity include female gender, diabetes, prior CVDs, and age [5, 6].

According to GLOBOCAN 2020, lung cancer alone accounted for an estimated 1.8 million deaths (18%), making it the leading cause of cancer mortality [3]. A significant 85% of these cases are diagnosed as non-small cell lung cancer (NSCLC) [7]. Although treatments for lung cancer have advanced significantly, particularly in the realm of chemotherapy [8], the cardiotoxicity associated with targeted drug therapies warrants vigilant monitoring [9, 10]. Predicting the onset of cardiotoxicity remains a challenge [11]. In drug research, co-culture systems are valuable as they allow for the comprehensive examination of drug effects on cell-to-cell interactions [12, 13]. Two drugs commonly utilized in lung cancer treatments, vinorelbine and carboplatin, have shown notable improvements in disease-specific survival. Specifically, vinorelbine, a semi-synthetic vinca alkaloid, exhibits anti-cancer properties, especially against advanced NSCLC [14–16]. Carboplatin, part of the platinum-based anti-cancer drug family, is considered standard care for advanced NSCLC when used in combination therapies [17, 18].

MiRNAs, single-stranded non-coding RNAs consisting of about 22 nucleotides, play pivotal roles in regulating gene expression. They can be used as therapeutic targets in CVDs, influencing various cardiac functions [19, 20], and also play key roles in NSCLC development [21, 22]. miRNA modulation was proposed as a promising therapeutic strategy to prevent the cardiotoxicity induced by different oncologic treatments, such as chemotherapy, radiotherapy, and ionizing radiation [23]. Changes in miRNA expression correlate with the onset and progression of cardiac diseases [24, 25]. Their profound modulatory effects on signaling pathways position miRNAs as potential biomarkers for cardiotoxicity and as therapeutic targets [23]. In this study, we focus on a panel of three miRNAs: miR-205-5p, miR-21-5p, and miR-30a-5p. These are among the miRNAs modulated by anticancer treatments known to influence cardiotoxicity.

## Materials and methods

### Cells and reagents

Ventricular normal human cardiac fibroblasts (NHCF-V) were procured from Lonza (NHCF-V; #CC-2904, Lonza, Basel, Switzerland) and maintained in Fibroblast Growth Medium-3 supplemented with 10% fetal bovine serum (FBS) (FGM3; #CC-4526, Lonza, Basel, Switzerland). Two lung adenocarcinoma cell lines (LUAD), namely, H1792 and Calu6, were obtained from the American Type Culture Collection (ATCC, Manassas, VA, USA). The H1792 cells were cultured in RPMI-1640 cell culture medium (Gibco, Thermo Fischer Scientific, USA) enriched with 10% FBS, 1% Glutamine, and 1% Penicillin, all sourced from Gibco (Grand Island, NY, USA). Conversely, Calu6 cells were maintained in MEM cell culture medium (Gibco, Thermo Fischer Scientific, USA), supplemented with 10% FBS and 1% Penicillin. All cultured cells were housed in a humidified incubator set at 37 ^∘^C with 5% CO_2_. Initially, each cell line was grown in its designated culture medium. Subsequently, all three cell lines were cultivated in the MEM medium. Treatments using 1 µM vinorelbine and 100 µM carboplatin were administered to all three cell lines for a duration of 48 h.

### Evaluation of cell invasion

Both lung cancer cell lines were seeded at a density of 15 × 10^4^ cells/well. After 24 h, cells were subjected to both single and combined treatments. At the 48-h mark, mitomycin was introduced, and a scratch was made at the bottom of the well using a 20 µL pipette tip. Subsequently, the medium was refreshed.

Images were captured at 0, 24, 48, 72, and 96 h using an Olympus IX71 microscope (Shinjuku-ku, Tokyo, Japan) equipped with cellSens software (scale bar—200 µM). These images were further processed and analyzed using ImageJ Software (version 2.0, Madison, WI, USA).

### Apoptosis assay

Apoptosis was assessed using the Multi-Parameter Apoptosis Assay Kit (Cayman cat no 600330, Estonia) in accordance with the manufacturer’s protocol. The cells were plated at a density of 8 × 10^4^ cells in 96-well plates and incubated overnight at 37 ^∘^C. Following this, the previously mentioned treatments were applied 24 h later, and cells were incubated for an additional 48 h at 37 ^∘^C under 5% CO_2_. For evaluation of apoptosis, cells were double-stained with tetramethyl rhodamine ethyl ester (TMRE) dye was used to assess mitochondrial membrane activity potential, while Hoechst dye was utilized for nuclear staining.

### Evaluation of apoptosis using flow cytometry

To evaluate specific modifications that occurred in cell apoptosis, the cells were plated at the density of 1 × 10^5^ in 24-well plates. The treatment with 1 µM vinorelbine and 100 µM carboplatin was performed 24 h post incubation, followed by another 48 h at 37 ^∘^C. After 48 h, cells were trypsinized, incubated in 1X Binding buffer, and stained with Annexin-V FITC at 4 ^∘^C for 10 min. A wash with 1X PBS followed the incubation to remove the excess dye, and the cellular pellet was resuspended in 1X Binding buffer. Before the acquisition, 2 µL of propidium iodide (PI) was added. BD FACS Canto II (San Jose, CA, USA) was used to obtain the cells, and FACS Diva software version 6.0 (San Jose, CA, USA) was used to perform the analysis.

### Evaluation of cell cycle using Celigo image cytometry

The cells were plated in a number of 10,000 cells/well in a final volume of 100 µL/well and incubated at 37 ^∘^C and 5% CO_2_ for 24 h. After 24 h, cells were treated with single and combined treatment. Forty-eight hours after treatment, media was removed, 100 µL ice-cold 100% methanol was added, and the plate was incubated for 30 min at 4 ^∘^C. After incubation, methanol was aspirated and replaced with PI working solution (1–2 µg/mL) with DNase-free RNase. After 45 min of incubation at 37 ^∘^C, the PI staining solution was removed, one wash was performed with PBS, and finally added 200 µL PBS. The plate was read at Celigo in Target 1 + 2.

### Autophagy detection

Autophagy effects were evaluated with Olympus IX71 inverted microscope (Shinjuku-ku, Tokyo, Japan) using Autophagy/Cytotoxicity Dual Staining Kit (Cayman cat no. 600140) according to the manufacturer’s protocol. Thus, cells were plated at a density of 8 × 10^4^ in a 96-well plate and treated with the abovementioned concentrations. Forty-eight hours after therapy, autophagic vacuoles were stained with monodansylcadaverine (MDC) and observed under UV light. Cell death was assessed using PI at 520/610 nm.

### Evaluation of miRNAs by real-time quantitative PCR

NHCF-V and H1792 cells, as well as NHCF-V and Calu6 cells, were seeded at a density of 3 × 10^5^ cells per well. Seeding was done in Corning™ Transwell™ multi-well plates equipped with permeable polycarbonate membrane inserts (6 wells, 0.4 µm pore size) containing 2 mL of complete media. The NHCF-V was positioned on the basal side of the plate, whereas lung cancer cells were situated on the apical side. Following a 24-h incubation, both basal and apical cells underwent treatment with the compounds of interest, either individually or in combination.

After another 48 h, cells were harvested in Trizol and used further for RT-PCR assay. RNA extraction was accomplished via the Phenol–Chloroform method. The extracted RNA was quantified using a NanoDrop spectrophotometer (ThermoFischer Scientific, Waltham, MA, USA). From this, 50 ng of total RNA was reverse transcribed to cDNA using the TaqMan MicroRNA Transcription kit (ThermoFischer Scientific, Waltham, MA, USA) and the TaqMan microRNA primer assay (ThermoFischer Scientific, Waltham, MA, USA) for the targeted miRNAs: miR-21-5p, miR-30c-5p, and miR-205-5p. The primer sequences for the selected miRNAs were as follows: hsa-miR-205-5p: UCCUUCAUUCCACCGGAGUCUG, hsa-miR-21-5p: UAGCUUAUCAGACUGAUGUUGA, hsa-miR-30a-5p: UGUAAACAUCCUCGACUGGAAG. The TaqMan Master Mix (Thermo Fisher Scientific) was utilized for miRNA evaluation, with RT-qPCR carried out on the ViiATM7 System (Applied Biosystems). Housekeeping miRNAs, RNU48, and U6, served as controls. The 2^−ΔΔCT^ method was employed for data analysis of the obtained CT values, and results were interpreted using GraphPad Prism software v.9 (GraphPad Software, San Diego, CA, USA).

### Western blotting analysis

After treatment, the cells were washed twice with PBS and then lysed in RIPA buffer (Coolaber, China). The samples were subsequently sonicated, and insoluble debris was removed by centrifugation at 13,000 rpm for 15 min. Protein content was determined using the BCA method (Coolaber, China). Thirty µg of each sample was separated on Mini Protean TGX gels (4%–20% acrylamide; Bio-Rad Laboratories). Electrophoresis was performed continuously at 125 V for 1 h. The gels were equilibrated in transfer buffer (25 mM Tris base, 192 mM glycine, and 10% methanol) for 30 min. Proteins were then transferred to PVDF membranes (Bio-Rad Laboratories) at 25 V, 1.0 A for 30 min. The membranes were blocked using Tris-buffered saline (TBS; 20 mM Tris base, 137 mM NaCl, pH 7.6) containing 3% BSA for 1 h. Primary antibodies were diluted in TBS supplemented with 0.1% Tween-20 and 3% BSA and were incubated overnight at 4 ^∘^C. The next day, membranes were washed, followed by a 1 h incubation with horseradish peroxidase-conjugated secondary antibodies. Membranes were subsequently washed in TBS-T. Finally, they were incubated with Clarity Western ECL Substrate (Bio-Rad Laboratories) for 5 min, enabling band detection using the Azure c300 digital imager system (Azure Biosystems, Dublin, CA, USA). For immunoblotting, primary antibodies against beta-Actin (#MAB8929, R&D, 0.01 µg/mL), p53 (#AF1355, R&D, 0.25 µg/mL), and BCL-2 (#15071, Cell Signaling, dil 1:1000) were used. Secondary antibodies, diluted at 1:1000, were purchased from Cell Signaling.

### Statistical analysis

The resulting data were expressed as mean ± standard deviation (SD). A *t*-test was used to determine the differences between experimental conditions, with *P* < 0.05 considered statistically significant. Statistical analyses were conducted using GraphPad Prism software (version 8.0; GraphPad Software, Inc., San Diego, CA, USA). The miRNet (https://www.mirnet.ca) database [26] was utilized to identify miRNAs as potential biomarkers.

## Results

### Antiproliferative effects of vinorelbine and carboplatin treatments on NHCF-V, Calu6, and H1792 cell lines

The initial phase of our study involved functional tests in a monoculture system, assessing the effects of the selected drugs on both tumoral and normal cells. Treatment was performed with a dose of 1 µM for vinorelbine and 100 µM for carboplatin at 48 h post-treatment to evaluate antiproliferative effects.

### Vinorelbine and carboplatin treatment-induced apoptosis inNSCLC cells and cardiomyocytes

**Figure 1.**
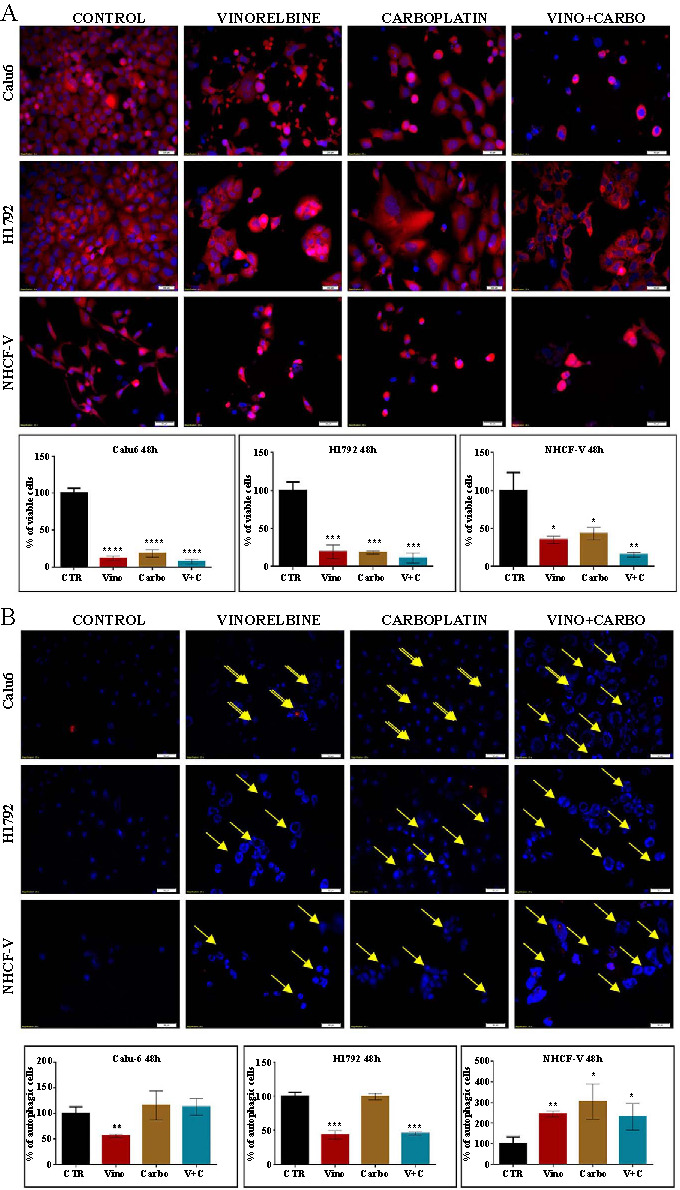
**Apoptosis evaluation for the vinorelbine and carboplatin alone or the combined therapy.** (A) Data presented as mean ± SD, *P < 0.05, **P < 0.01, ***P < 0.001, ****P < 0.0001, two-sided *t*-test; the evaluation of apoptosis through fluorescence microscopy (TMRE and Hoechst staining) following exposure to 1 µM vinorelbine and 100 µM carboplatin, and the combined therapy, 1 µM vinorelbine + 100 µM carboplatin, on NHCF-V and lung cancer cells in triplicate (20× magnification), TMRE-Hoechst staining; (B) Data presented as mean ± SD, *P < 0.05, **P < 0.01, ***P < 0.001, ****P < 0.0001, two-sided *t*-test; the evaluation of autophagy (MDC staining) through fluorescence microscopy following exposure to 1 µM vinorelbine and 100 µM carboplatin, and the combined therapy, 1 µM vinorelbine + 100 µM carboplatin, on NHCF-V and lung cancer cells (20× magnification), MDC/PI staining; TMRE: Tetramethyl rhodamine ethyl ester; MDC: Monodansylcadaverine; SD: Standard deviation; NHCF-V: Ventricular normal human cardiac fibroblast.

To understand the impact of both individual and combined treatments on the progression of lung cancer cells, we performed functional analyses on the Calu6, H1792, and NHCF-V cell lines. Using fluorescence microscopy 48 h post-treatment, we assessed apoptotic effects via double staining with TMRE and Hoechst. As depicted in Figure 1, both the NHCF-V and lung cancer cells displayed an undisrupted mitochondrial membrane potential. However, after 48 h of therapy (with vinorelbine, carboplatin, or their combination), there were significant alterations in the cellular morphology and a reduction in the number of viable NHCF-V and lung cancer cells. This therapy significantly reduced the mitochondrial membrane potential in the lung cancer cells, though NHCF-V cells maintained their undisrupted mitochondrial membrane potential. Additionally, Hoechst staining revealed irregular and fragmented nuclei.

Fragmented nuclei were markedly evident post-treatment, showcasing the effects of the treatments relative to the control group (Figure 1A). The combined therapy induced an increased PI staining compared to control cells, suggesting that late apoptosis or necrosis is induced by the selected compounds at a significantly higher rate than in the control conditions (Figure 1B). The presence of autophagic vacuoles was revealed through MDC accumulation, presenting as dot-like structures that rendered the cells a faded blue hue.

**Figure 2.**
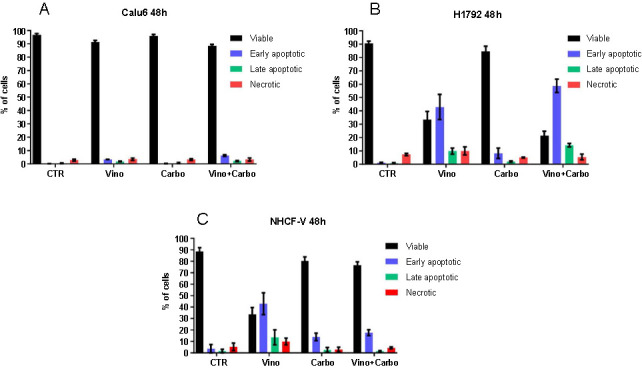
**Apoptosis evaluation after 48 h of treatment with 1 µM vinorelbine, 100 µM carboplatin and combined therapy, 1 µM vinorelbine and 100 µM carboplatin, using flow cytometry**. The samples were evaluated in triplicate. Cells were double-stained with annexin-V FITC and PI. (A) Apoptotic cell percentage in the Calu6 population; (B) Apoptotic cell percentage in the H1792 population; (C) Apoptotic cell percentage in the NHCF-V population. NHCF-V: Ventricular normal human cardiac fibroblast; PI: Propidium iodide; FITC: Fluorescein isothiocyanate; Vino: Vinorelbine; Carbo: Carboplatine; CTR: Combined therapy.

Subsequent flow cytometry confirmed apoptosis activation. For the Calu6 cell line treated with vinorelbine and combined therapy, there was a rise in the percentage of apoptotic cells in both early and late stages. A decrease in viable cells was also identified compared to the control groups. In contrast, the carboplatin treatment showed no significant effects relative to control cells (Figure 2A). For the H1792 cells, a decrease in viable cells was evident post-vinorelbine and combined treatments. Furthermore, an increase in the number of apoptotic cells in early and late apoptosis. With carboplatin therapy, the number of viable cells marginally reduced, with some entering early-stage apoptosis (Figure 2B). Thus, both individual and combined therapies greatly influenced the nuclear morphology of the H1792 cell line, indicating apoptosis.

### Cell cycle assessment for cardiomyocytes and NSCLC cells treated with vinorelbine and carboplatin

**Figure 3.**
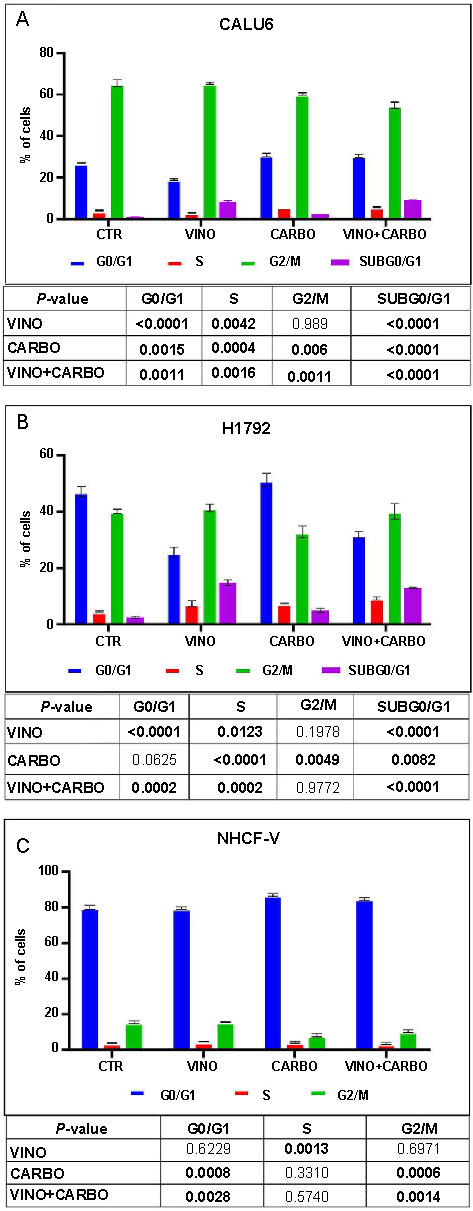
**Cell cycle evaluation after 48 h of treatment with 1 µM vinorelbine, 100 µM carboplatin, and combined therapy.** The samples were evaluated in triplicate. Cells were stained with propidium iodide. (A) The cell cycle phases in the Calu6 population; (B) The cell cycle phases in the H1792 population; (C) The cell cycle phases in the NHCF-V population. NHCF-V: Ventricular normal human cardiac fibroblast; VINO: Vinorelbine; CARBO: Carboplatin; CTR: Combined therapy.

We observed cell cycle alterations in the Calu6 and H1792 cell lines 48 h after treatment with 1 µM vinorelbine, 100 µM carboplatin, and their combination (Figure 3). As can be observed, a higher sub-G0/G1 population was identified in the Calu6 cell line treated with vinorelbine and the combination therapy, demonstrating the presence of apoptotic cells. Furthermore, the number of cells in the S phase was higher compared to the control groups when treated with carboplatin and the combination therapy (Figure 3A). For H1792 cells, both the sub-G0/G1 and S phase populations were significantly increased across all treatments when compared to the controls (Figure 3B). Meanwhile, for cardiomyocytes, there was an increase in the G0/G1 phase cells post carboplatin and combined therapy treatment, whereas cells in the G2/M phase decreased with these therapies (Figure 3C).

### Wound healing assay to evaluate the effects of vinorelbine and carboplatin on NSCLC cell migration

**Figure 4.**
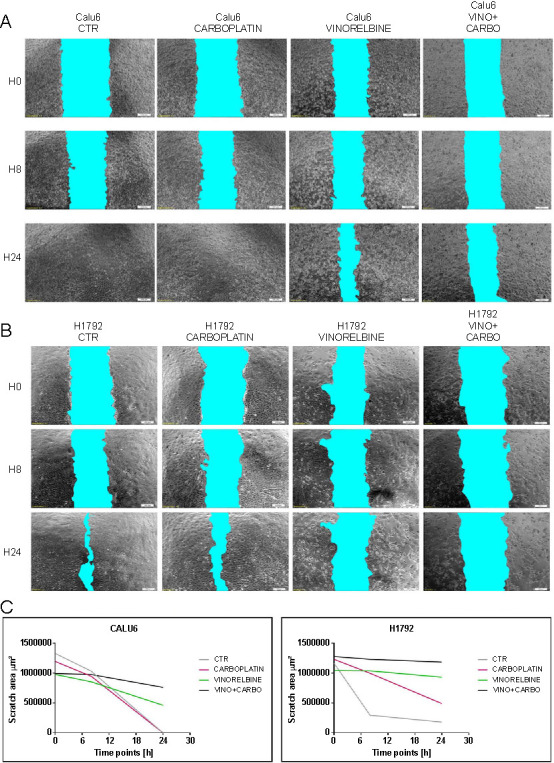
**Representative images of cell migration during wound healing.** The wound healing assay was performed on Calu6 and H1792 cell lines in triplicate 48 h post-therapy with 1 µM vinorelbine and 100 µM carboplatin; (A) Calu6 cells; (B) H1792 cells; (C) Quantitative graphical representation of the wound distance measurement in µm related to the treatment time. VINO: Vinorelbine; CARBO: Carboplatine; CTR: Combined therapy.

Cell migration was evaluated after 48 h using 1 µM vinorelbine and 100 µM carboplatin, both lung cancer cell lines displayed varied migration compared to their controls. Notably, the Calu6 cells treated with carboplatin had their wound close within 24 h of scratching, whereas those treated with vinorelbine and the combined therapy had wounds still visible after this period (Figure 4A). Figure 4B indicates that the gap-closing rate for the vinorelbine and combined therapy mirrored the initial gap seen 8 h post-scratch, highlighting vinorelbine’s pronounced effect on cell migration and invasion. For H1792 cells, carboplatin had a milder effect on migration compared to both vinorelbine and the combination treatment.

### miRNAs exhibited differential expression patterns in a co-culture system of cardiomyocytes with NSCLC cell lines after vinorelbine and carboplatin therapy

**Figure 5.**
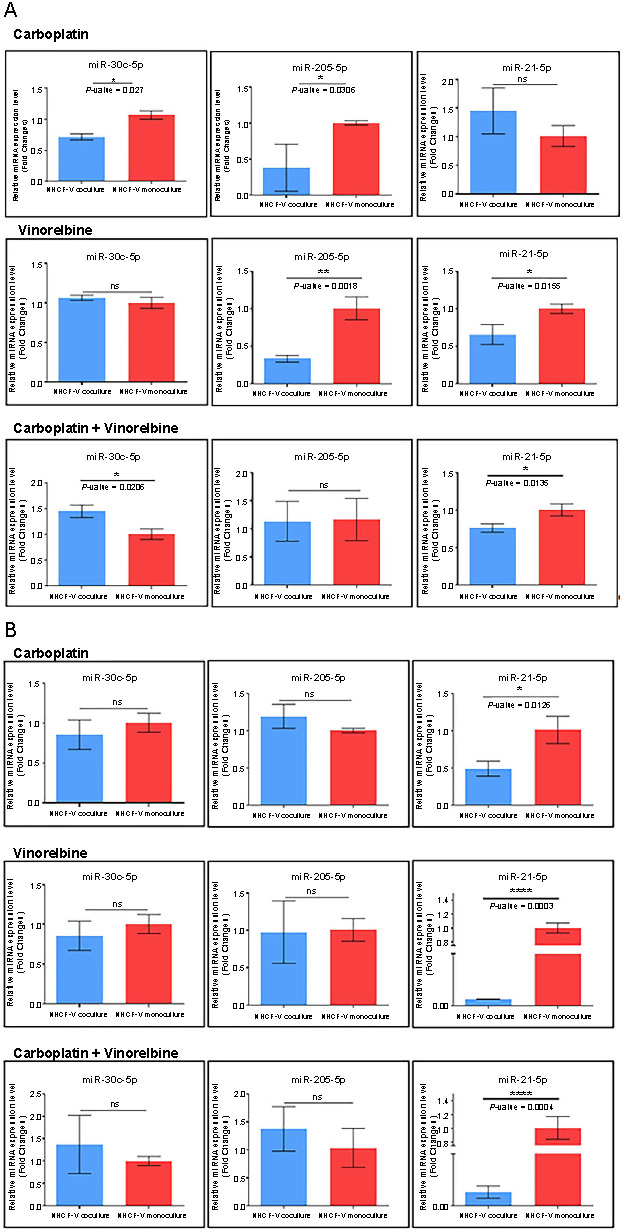
**The evaluation of the effect of 1 µM vinorelbine and 100 µM carboplatin on NHCF-V cells in triplicate from monoculture and co-culture.** The data were normalized to U6 and RNU48 using the 2^−ΔΔCT^ method. A two-sided *t*-test was used to analyze the data where *P < 0.05, **P < 0.01, ***P < 0.001, ****P < 0.0001, with (A) Calu6 and (B) H1792 on selected miRNAs. Relative miRNA expression levels are shown for miR-30c, miR-21, and miR-205. NHCF-V: Ventricular normal human cardiac fibroblast.

In this segment, we examined the roles of miR-21-5p, miR-30c-5p, and miR-205-5p in a co-culture system of cardiomyocytes with NSCLC cells. Figure 5 demonstrates varying expression patterns for these miRNAs across three treatment scenarios.

Carboplatin Scenario: Cells treated with 100 µM carboplatin in a co-culture system (NHCF-V cells on the basolateral side of the insert, Calu6 cells cultured on the apical side) compared to NHCF-V monocultured with 100 µM carboplatin in a monolayer).

Vinorelbine Scenario: Cells treated with 1 µM vinorelbine using co-culture system (NHCF-V cells on the basolateral side of the insert and Calu6 cells cultured on the apical side) vs NHCF-V monocultured with 1 µM vinorelbine in a monolayer.

Combined Treatment Scenario: Vinorelbine + carboplatin cells treated with 1 µM vinorelbine + 100 µM carboplatin using co-culture system (NHCF-V cells on the basolateral side of the insert and Calu6 cells cultured on the apical side) vs NHCF-V monocultured with 1 µM vinorelbine + 100 µM carboplatin in a monolayer.

Figure 5A portrays the expression levels of these miRNAs in the Calu6 co-culture system (NHCF-V and Calu6 cell lines) compared to the monoculture system (NHCF-V cells). In the case of miR-30c, we observed a significant decrease in the expression profile in the first scenario (*P ═ 0.0278) and a significant increase in the third scenario (*P ═ 0.0206), while, in the second scenario, the expression level was slightly decreased but not significant. For miR-21, the expression level in the second and the third scenarios was downregulated (*P ═ 0.0155 and *P ═ 0.0135, respectively). In the first scenario, the expression level was upregulated but not statistically significant. miR-205, for the first and the second scenarios, showed decreased expression profile (*P ═ 0.0305 and **P ═ 0.0018, respectively) compared to the control, while in the third scenario, no significant alterations were exhibited compared to the control group (Figure 5A).

In Figure 5B, we observed the expression profile for selected miRNAs in the co-culture system (NHCF-V and H1792 cell lines) compared to the monoculture system (NHCF-V cells). The expression level of miR-30c was downregulated in the first and the second scenarios and upregulated in the third scenario. The expression level miR-21 in all three scenarios was significantly downregulated (*P ═ 0.0126, ****P ═ 0.0003, and ****P ═ 0.0004, respectively) compared to the monoculture system. In the case of miR-205, the first and the third scenarios exhibited a slight upregulation in their expression profile compared to the control. The expression level of miR-205 for the second scenario presents no significant alterations compared to the control group (Figure 5B).

### Differential expression patterns of miRNAs in a co-culture system of NSCLC cell lines with cardiomyocytes after vinorelbine and carboplatin treatment

**Figure 6.**
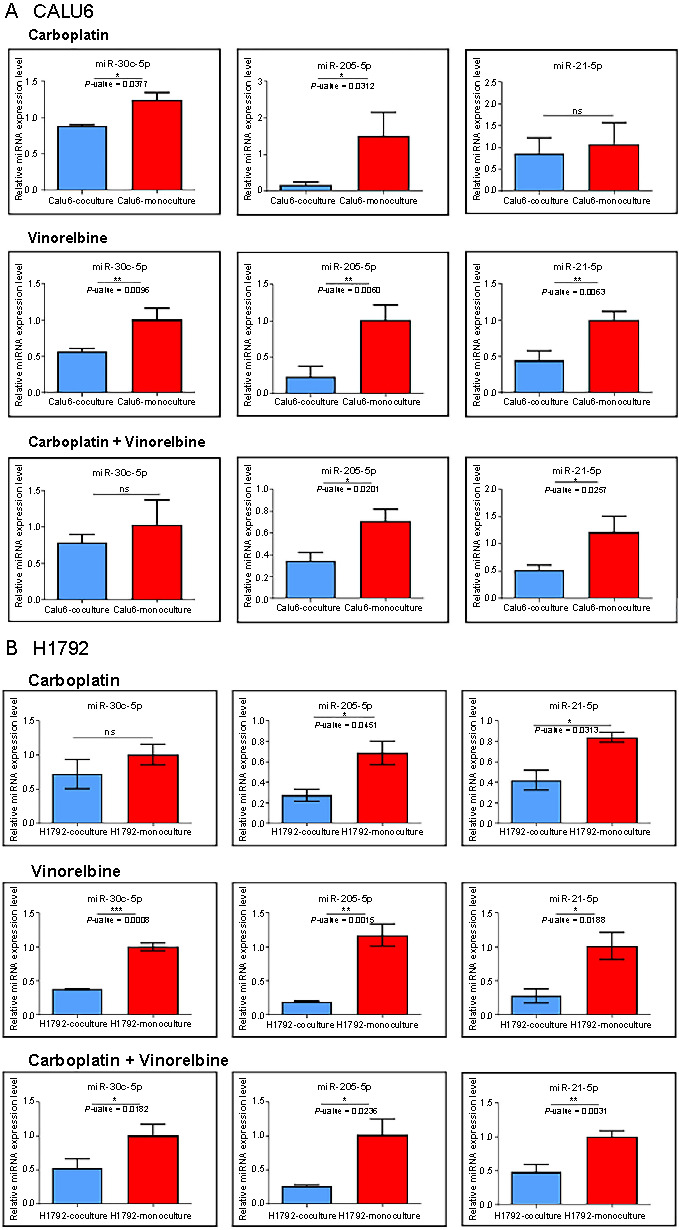
**The evaluation of the effect of 1 µM vinorelbine and 100 µM carboplatin.** The data were normalized to U6 and RNU48 using the 2^−ΔΔCT^ method. A two-sided *t*-test was used to analyze the data where *P < 0.05, **P < 0.01, ***P < 0.001, ****P < 0.0001, on (A) Calu6 and (B) H1792 cells in triplicate from monoculture and co-culture with NHCF-V cells on selected miRNAs. Relative miRNA expression levels are shown for miR-30c, miR-21, and miR-205.

In Figure 6A, we presented the expression level for miR-21-5p, miR-30c-5p, and miR-205-5p in a co-culture system (Calu6 and NHCF-V cell lines) compared to the monoculture system (Calu6 cells). For tumor cells, the expression profile for miR-21-5p, miR-30c-5p, and miR-205-5p also differs between the selected scenarios. Three different scenarios were investigated for each lung cancer cell line. First, carboplatin cells treated with 100 µM carboplatin using a co-culture system (tumoral cells on the apical side of the insert and NHCF cells cultured on the basolateral side) vs tumoral cell monoculture (100 µM carboplatin in a monolayer). Second, vinorelbine cells treated with 1 µM vinorelbine using co-culture system (tumoral cells on the apical side of the insert and NHCF cells cultured on the basolateral side) vs tumoral monoculture (1 µM vinorelbine in a monolayer). Third, vinorelbine + carboplatin cells treated with 1 µM vinorelbine + 100 µM carboplatin using co-culture system (tumoral cells on the apical side of the insert and NHCF cells cultured on the basolateral side) vs tumoral cells monoculture (1 µM vinorelbine + 100 µM carboplatin in a monolayer). For miR-30c, we identified a significant decrease in the expression profile of the first scenario (*P ═ 0.0377) and the second scenario (**P ═ 0.0095). In the third scenario, the expression level was slightly decreased but insignificant. miR-205-5p exhibited a significantly reduced expression profile for all three scenarios (*P ═ 0.0312, **P ═ 0.0060, and *P ═ 0.0201). The expression level was downregulated in the second and the third scenarios (**P ═ 0.0063 and *P ═ 0.0257) in the case of miR-21-5p. No significant alteration was observed for the first scenario.

The expression profile of the selected miRNAs for H1792 cells in the co-culture system compared to the monoculture system is shown in Figure 6B. Thus, miR30c-5p was significantly downregulated for the second and the third scenarios (***P ═ 0.0008 and *P ═ 0.0182, respectively) compared to the monoculture system. The expression level of miR-205-5p significantly decreased for all three scenarios (*P ═ 0.0451, **P ═ 0.0015, and *P ═ 0.0236, respectively). The same situation was observed for miR-21-5p (*P ═ 0.0313, *P ═ 0.0188, and **P ═ 0.0031, respectively).

### miR-30c-5p, miR-21-5p, miR-205-5p network interaction

**Figure 7.**
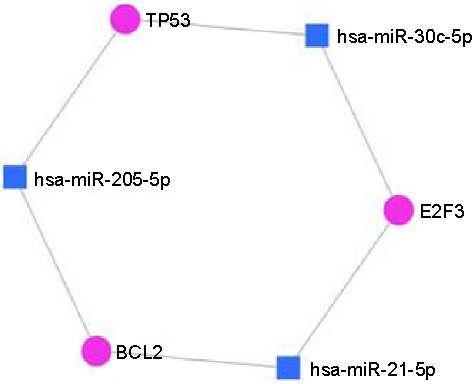
The miR-205–miR-30c–miR-21 mRNA network generated using miRNet to show the interaction network between the genes of interest and specific miRNAs.

Using miR-30c-5p, miR-21-5p, and miR-205-5p, we established miRNA–mRNA regulatory networks and explored their potential functions and interconnection with pivotal genes implicated in cancer (Figure 7).

### p53 and BCL2 protein assessment by western blot

**Figure 8.**
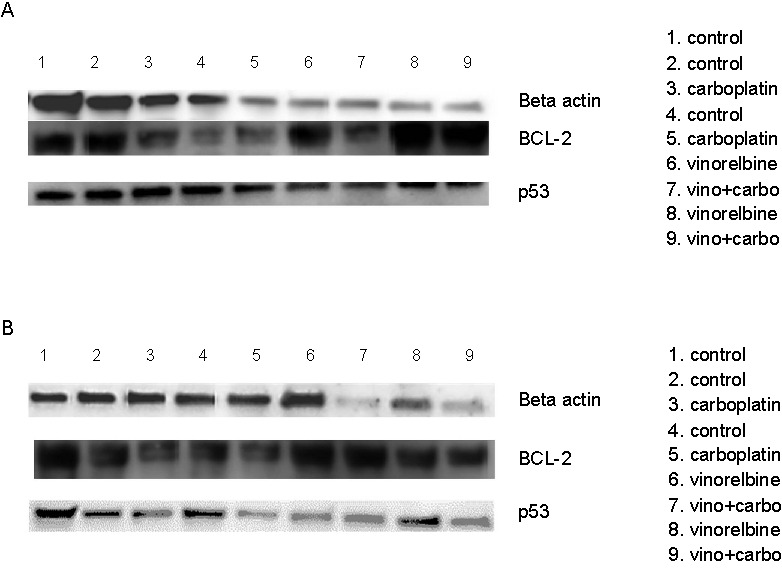
**Western blotting images of BCL-2 and p53 in co-culture of NHCF-V cells.** With (A) Calu6 and (B) H1792 in the context of 1 µM vinorelbine, 100 µM carboplatin and combined therapy. β-actin was used as a protein loading control.

The influence of carboplatin and vinorelbine on apoptosis, proliferation, and cardiotoxicity was assessed by co-culturing two lung cancer cell lines (H1792 and Calu6) with NHCF-V. Treatments led to the upregulation of p53 and BCL-2 proteins. Notably, the combination therapy exhibited a synergistic effect, as reflected in the heightened protein expression when compared to control groups (Figure 8).

## Discussion

This study investigated the cardiotoxic effects of carboplatin and vinorelbine in vitro using a co-culture system consisting of cardiomyocytes and two lung cancer cell lines. These two drugs operate via different mechanisms: carboplatin primarily targets DNA, inhibiting its replication and transcription, thereby inducing cell death [27]. On the other hand, vinorelbine is an anti-microtubule agent that triggers apoptosis in cancer cells by reducing the formation of heterodimers between Bcl2 and the pro-apoptotic gene BAX [28].

Secondary cardiac diseases have been identified as the leading cause of death among cancer survivors. This highlights the need for the development of novel therapeutic strategies aimed at mitigating the cardiotoxicity induced by anticancer drugs [29]. The administration of metronomic oral vinorelbine alone has been found to be safe for elderly patients with advanced NSCLC [30, 31].

Carboplatin has been shown to induce cardiotoxicity through a mitochondria-dependent apoptosis mechanism related to reactive oxygen species (ROS) production [32]. About 1% of patients treated with vinorelbine in clinical trials exhibited secondary cardiac diseases. However, existing evidence suggests that the side effects of vinorelbine are comparable to those of other chemotherapeutic agents with similar indications [33]. The combination of vinorelbine and carboplatin has been effective in first-line treatments for advanced NSCLC [34].

The co-culture model was employed to study the potential cardiotoxic and pro-inflammatory effects of chemotherapy. Co-culture serves as an effective model for monitoring drug-induced effects on cell–cell interactions. For instance, a cardiotoxic effect manifested as reduced viability of cardiomyocytes has been observed in co-cultures of tumor cells or human fetal cardiomyocytes with lymphocytes treated with pembrolizumab and trastuzumab [12].

Co-culturing murine and human embryonic stem-cell-derived cardiomyocytes with endothelial cells has been shown to enhance their maturity and upregulate several microRNAs [35].

miR-205 could regulate cardiac ischemia/reperfusion injury, demonstrating that low expression of miR-205 reduced oxidative stress in hypoxia/reoxygenation-treated cardiomyocytes [36]. miR-205 was found to be overexpressed in lung cancer tumor tissues compared to adjacent controls [22]. In our study, miR-205 was overexpressed in cardiomyocytes from a co-culture system with Calu6 cells following treatment with carboplatin and vinorelbine separately, but not when combined. In the co-culture system with H1792 cells, overexpression of miR-205 was observed for both carboplatin and its combination with vinorelbine.

In cardiomyocytes, cardiac fibroblasts, and conditioned medium derived from cardiomyocytes, miR-21 was found upregulated following oxygen–glucose-deprivation treatment, highlighting its important role in intercellular communication among ischemic cardiac cells [37]. A comparative study between chronic DOX-injury mice and acute DOX-injury mice indicated that miR-21 expression significantly increased after DOX treatment in both mouse heart tissue and H9C2 cells exposed to varying concentrations of DOX [38]. Our study revealed that miR-21 was overexpressed in cardiomyocytes from a co-culture system with Calu6 cell lines treated with vinorelbine and a combination of carboplatin and vinorelbine. Significant overexpression of miR-21 was also observed in cardiomyocytes from a co-culture system with H1792 cells for all treatment scenarios.

Our findings report significant overexpression of miR-30c in cardiomyocytes from a co-culture system with Calu6 cells treated with carboplatin, and a substantial decrease in expression levels with the combination treatment, the same as for the co-culture system with H1792 cells. In NSCLC patients treated with bevacizumab, an increase in serum miR-30c levels was linked to cardiotoxicity [38]. The impact of chemotherapeutic agents carboplatin and vinorelbine, and their combination, on apoptosis and proliferation was assessed using BCL2 and p53 protein expression as surrogate markers. In co-culture models of cardiomyocytes (NHCF-V) with NSCLC cell lines (H1792 and Calu6), both BCL2 and p53 protein expressions were upregulated as shown in western blot analyses (Figure 8). The combination of these drugs had a synergistic effect on BCL2 and p53 protein expression, suggesting combined cardiotoxicity.

Bcl-2 is an anti-apoptotic protein that plays a key role in regulating programmed cell death (apoptosis) by preventing the activation of caspases. Increased BCL2 expression has been linked to chemotherapy resistance and an elevated risk of cardiotoxicity in patients treated with anthracyclines. It serves as an adaptive mechanism in response to chemotherapeutic drugs and is also considered a marker of cardiotoxicity.

p53 is a tumor suppressor protein widely known as the guardian of the cell cycle, playing an essential role in regulating cell cycle progression and DNA repair in response to cellular stress. Elevated p53 levels can be attributed to the upregulation of the DNA damage response pathway in reaction to cellular damage induced by treatment regimens. Accumulation of p53 is a cellular response to increased oxidative stress and DNA damage, which is associated with increased cardiotoxicity from these agents [39, 40]. In a related experiment involving cardiomyocytes treated with doxorubicin, elevated p53 levels were shown to induce apoptosis, supporting the notion that high upregulation of p53 protein will block the cell cycle and lead to cellular death, thereby explaining the cardiotoxic effect [41].

## Conclusion

The current data support the utility of miRNAs, specifically miR-21-5p, miR-30c-5p, and miR-205-5p, as biomarkers for chemotherapy-induced cardiotoxicity. While the upregulation of Bcl-2 and p53 in response to carboplatin and vinorelbine treatment may be beneficial for cancer cell survival and therapeutic responsiveness, it also elevates the risk of cardiotoxicity.

Our study managed to advance the understanding regarding the influence of two widely used chemotherapeutic agents, carboplatin and vinorelbine, on cardiotoxicity. We utilized co-culture models and functional assays to achieve these insights. This study highlights the role of miRNAs and the dysregulation of anti-apoptotic proteins as key mechanisms underlying chemotherapy-induced cardiotoxicity. Further research is warranted to elucidate these mechanisms in greater detail and to identify potential strategies for mitigating the cardiotoxic effects of these chemotherapy drugs.
